# Effect of Day 3 cell number on the live birth rate of vitrified-warmed Day 5 single blastocyst transfer in young women

**DOI:** 10.1186/s12884-024-06468-1

**Published:** 2024-04-19

**Authors:** Pingping Qiu, Ronghui Ye, Ping Li, Hui Huang, Lu Ding

**Affiliations:** 1 Women and Children’s Hospital, School of Medicine, Xiamen University, Xiamen, 361000 Fujian China; 2 Xiamen Key Laboratory of Reproduction and Genetics, Xiamen, 361000 Fujian China; 3 Xiamen Assisted Reproductive Technology Quality Control Center, Xiamen, 361000 Fujian China

**Keywords:** Day 3 cell number, Single blastocyst transfer, Vitrified-warmed cycles, Live birth rate

## Abstract

**Background:**

Previous studies have reported inconsistent results regarding blastocyst selection with a high day 3 (D3) cell number and the eventual pregnancy outcomes. Thus, in this study, the relationship between the D3 cell number and clinical outcomes of day 5 single blastocyst transfer (SBT) in vitrified-warmed transfer cycles was investigated.

**Methods:**

Our retrospective study included 1144 day 5 SBT in vitrified-warmed cycles between February 2016 and February 2021. All cycles were the first vitrified-warmed cycles, and the female patients were less than 35 years of age. Based on the D3 cell number, the cycles were divided into four groups, as follows: group A (3–7 cells, *n* = 130); group B (8–9 cells, *n* = 621); group C (10–12 cells, *n* = 328); and group D (13–16 cells, *n* = 65). The differences in the live birth rate (LBR), clinical pregnancy rate, and miscarriage rate were examined among the four groups.

**Results:**

The LBR and clinical pregnancy rate increased with the D3 cell number (*P* < 0.01). No significant difference was found in the miscarriage rate among the groups (*P* = 0.055). After adjusting for confounding factors, the LBR was significantly higher in groups C (odds ratio [OR] = 1.477, 95% confidence interval [CI]: 1.124–1.941, *P* = 0.005) and D (OR = 2.000, 95% CI: 1.166–3.429, *P* = 0.012) than in group B.

**Conclusions:**

A high D3 cell number (> 9 cells) was associated with a high LBR in the vitrified-warmed day 5 SBT cycles of patients < 35 years of age. The cell number of D3 embryos can be an important reference indicator for blastocyst selection. Among blastocysts with the same morphological score, those with > 9 cells on D3 can be preferentially selected for transplantation.

## Background

In recent years, assisted reproductive technology (ART) has been widely used and has gradually improved. With the continuous optimization of various technologies, such as ovulation induction, blastocyst culture, and embryo cryopreservation and recovery, more institutions tend to perform selective single blastocyst transfer (SBT). Culturing cleavage embryos to the blastocyst stage and then transferring them allows for the selection of relatively viable embryos [1]. This stage is similar to the natural implantation time, and the endometrial and embryonic development can be comparatively synchronized [2]. Additionally, SBT can help reduce the multiple pregnancy rate and avoid adverse obstetric outcomes [1].

The Gardner grading system [3, 4] is currently used for blastocyst selection. The system is based on the qualitative description of blastocyst morphology rather than quantitative evaluation, which is subjective and has a limited capacity for blastocyst differentiation. In addition, most patients do not undergo preimplantation genetic testing before embryo transfer. Thus, selecting a blastocyst with the maximum development potential for transfer remains a major challenge.

The mainstream embryology theory states that the optimal blastomere number of day 3 (D3) embryos is 7–9 cells. Most of these embryos exhibit normal cleavage behavior and should be preferentially selected for transfer. Embryos with slow or fast cleavage rates are generally considered to be abnormally developed and have a high probability of chromosomal aneuploidy. However, some researchers have reported contrary results; they found that fast-cleaving embryos have a higher rate of high-quality blastocyst formation than slow and moderate-cleaving embryos. Hence, the high cell number on D3 is a good pregnancy outcome indicator for blastocyst selection [5].

To date, some studies have focused on the relationship between the D3 cell number and pregnancy outcomes, but their conclusions are inconsistent [6–10]. Thus, in this study, we retrospectively analyzed 1144 clinical cases to explore the impact of D3 cell number on the clinical outcomes of vitrified-warmed SBT cycles. The study findings are anticipated to help optimize the indicators and strategy used for blastocyst selection.

## Methods

### Study material

**Fig. 1:**
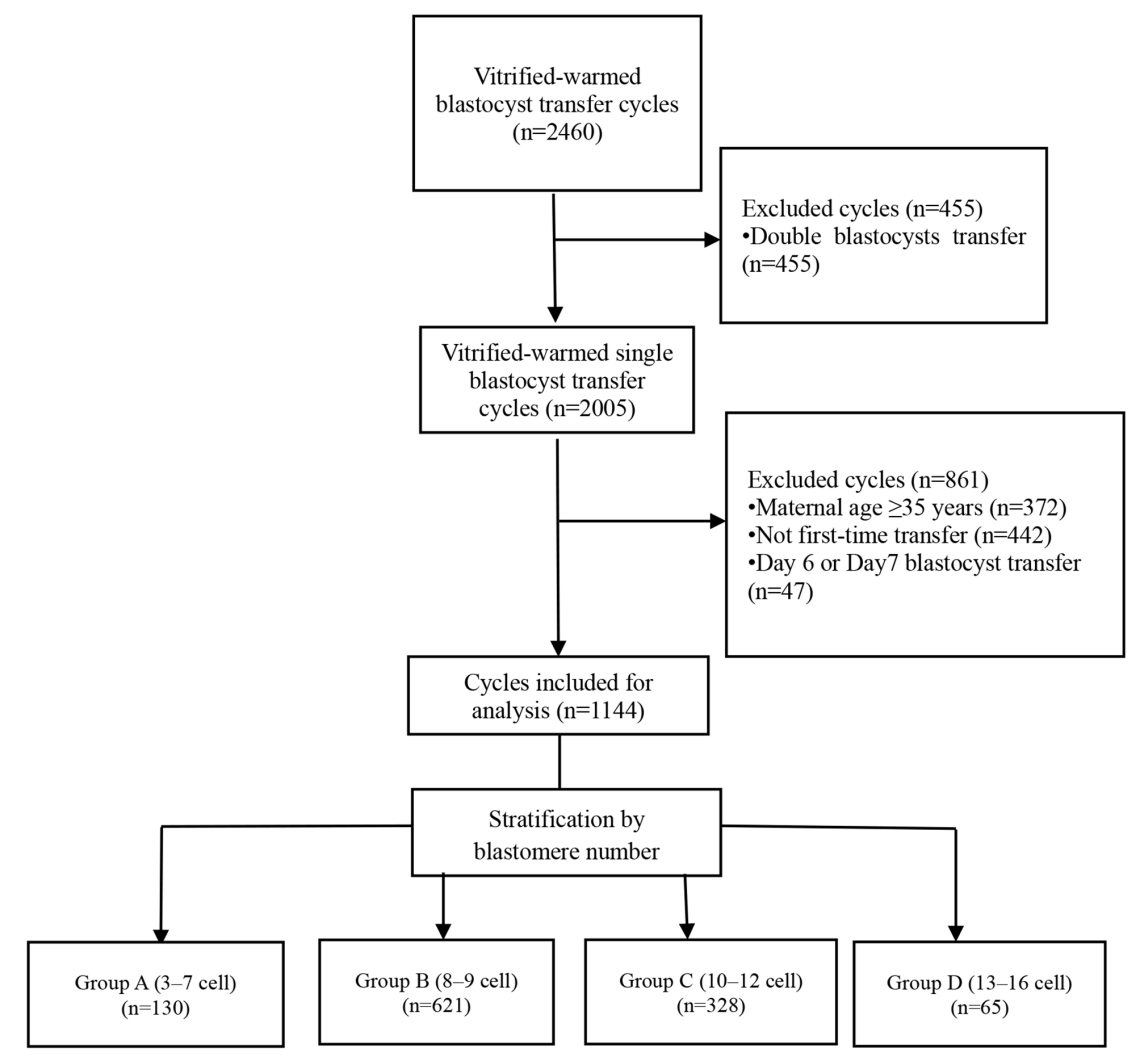
Scheme showing the study design

This retrospective study was conducted at the Department of Reproductive Medicine, Xiamen Women and Children’s Hospital, affiliated with Xiamen University. The inclusion criteria were as follows: maternal age < 35 years, first frozen-thawed transfer cycles, and single day 5 blastocyst transfer. The exclusion criteria included donor oocyte cycles, repeated implantation failure, acquired or congenital uterine abnormalities (such as congenital uterine malformations, intrauterine adhesions, endometrial polyps and submucosal fibroids, and severe adenomyosis) diagnosed using 3D ultrasound, a lack of core data, and lost cases. Based on these criteria, data on 1144 cycles between February 2016 and February 2021 were included (Fig. 1). These cycles were divided into four groups based on the number of cells in the transplanted blastocysts on D3, as follows: group A (3–7 cells), group B (8–9 cells), group C (10–12 cells), and group D (13–16 cells). This study was approved by the Ethics Committee of Xiamen Women and Children’s Hospital (Approval number: KY-2022-057-K01) and was performed in line with the Declaration of Helsinki. According to the Ethics Committee of Xiamen Women and Children’s Hospital, the requirement for informed consent was waived owing to the retrospective nature of the study, and data from all patients were used anonymously.

### Experimental protocols

The ovulation induction program was based on the routine program determined by the department. The agonist and antagonist programs were selected to stimulate ovulation based on the patients’ ovarian reserve, homogeneity of the basic antral follicle size, and the receptivity of the endometrium. When the majority of follicles reached 18–20 mm in diameter, 200–250 µg of recombinant human chorionic gonadotropin (Azer, Merck Serono, Fenil-sur-Corsier, Switzerland) was administered to trigger ovulation. After 36 h, under intravenous anesthesia and using ultrasound guidance, oocytes were retrieved through a vaginal puncture.

Fertilization was achieved using in vitro fertilization (IVF) or intracytoplasmic sperm injection (ICSI). Embryos were cultured using the G5 sequential medium (Vitrolife, Göteborg, Sweden) at 37 °C in 6% CO_2_, 5% O_2_, and 89% N_2_. Per the 2011 Istanbul Consensus [11], the number of blastomeres and embryo scores were recorded between 67 and 69 h after fertilization.

On day 3, the cleavage-stage embryos were transferred from G-1 PLUS medium to the G-2 PLUS medium for further culture. Both the cleavage embryo and blastocyst culture in our laboratory were subjected to microdroplet culture, and only one embryo was placed in each microdroplet. Each embryo had its own number (NO). According to the NO, the embryos were transferred into a microdroplet in a G2 Petri dish, one by one. The entire process was verified by two embryologists to ensure that the embryos were transferred in numerical order.

Blastocysts were scored according to the Gardner blastocyst scoring system [3, 4]. In our laboratory, blastocysts with inner cell mass (ICM) and trophectoderm (TE) scores of AA, BA, AB, and BB were defined as high-quality blastocysts, and the rest were considered low-quality blastocysts. Frozen blastocysts were selected if they were at stage 3 or above and their ICM score was not C.

Before vitrification, the blastocysts were shrunk using a laser to release the cystic fluid. Vitrification and warming were performed according to the instructions of the MediCult Vitrification Cooling and Warming Kit (Origio, Malov, Denmark). After warming, the blastocysts were transferred to a blastocyst culture medium and cultured for at least 2 h before transplantation. Blastocyst survival was determined based on the re-expansion of the blastocyst cavity.

The endometrial preparation protocol for the vitrified-warmed cycle was formulated per the standard operating procedures of our department. The natural cycle was adopted for patients with normal ovulation, and dydrogesterone (10 mg; Abbott, Abbott Park, IL, USA) was administered BID PO on the day after ovulation. The blastocysts were transferred 5 d after ovulation, and the dydrogesterone dosage was increased to 20 mg BID PO. The hormone replacement treatment (HRT) program was used to prepare the endometrium in recipients with irregular menstruation or no natural cycle ovulation. During this program, oral estradiol valerate (Bayer, Leverkusen, Germany) (4–9 mg) was administered daily. When the endometrial thickness was ≥ 8 mm, dydrogesterone (20 mg) was administered BID PO. After 5.5–6 d, blastocyst transfer was performed and progesterone vaginal gel (Crinone; Merck Serono) was administered 1 QD on the day of transplantation. Serum β-human chorionic gonadotropin level was measured 10–12 d after transplantation.

### Outcome assessment

The primary outcome recorded in this study was the live birth rate (LBR). Secondary endpoints included clinical pregnancy and miscarriage rates. Live birth was defined as the delivery of a viable infant after 28 weeks of gestation. Clinical pregnancy was confirmed by visualization of a gestational sac on ultrasound with or without cardiac pulsation 28–30 d after transplantation, and miscarriage was defined as a clinical pregnancy loss before the 24th gestational week.

### Statistical analyses

SPSS 28.0 software (IBM Corp., Armonk, NY, USA) was used for data analysis. A *post hoc* power analysis was performed using G-power. For multi-group measurement, data that conformed to a normal distribution were expressed as mean ± standard deviation. Measurement data that did not conform to a normal distribution were expressed as median [M (P_25_, P_75_)], and the median comparison was performed using the Kruskal–Wallis test. Rates (%) in enumeration data were compared using the adjusted χ² test or Fisher’s exact test. The Bonferroni correction was used for data analysis, and *P* < 0.008 (0.05/6) was considered statistically significant. A logistic regression model was used to analyze the effect of D3 cell number on LBR after adjusting for confounding factors. The odds ratios (ORs) with 95% confidence intervals (CIs) were calculated. Results with *P* < 0.05 were considered statistically significant.

## Results

A total of 1144 single blastocyst transplantation cycles were analyzed; 675 cycles resulted in a clinical intrauterine singleton pregnancy, and 563 infants were born. The clinical pregnancy rate and LBR were 59.0% and 49.2%, respectively. The average maternal age was 29.2 years (range: 20–34 years). The number of cycles with 3–7, 8–9, 10–12, and 13–16 cell embryos was 130 (11.3%), 621 (54.3%), 328 (28.7%), and 65 (5.7%), respectively. The baseline characteristics of the four groups are shown in Table 1. The rate of high-quality blastocysts was significantly lower in group A than in the other three groups (*P* < 0.008).

**Table 1:** Demographic characteristics of frozen-thawed SBT cycles grouped according to D3 cell numbers

Characteristics	Group A (*n* = 130)	Group B (*n* = 621)	Group C (*n* = 328)	Group D (*n* = 65)	*P* value
Maternal age(years), median (IQR)	30.0 (27.8, 32.0)	29.0 (27.0, 31.0)	29.0 (27.0, 31.0)	29.0 (28.0, 31.0)	0.182
Duration of infertility (years)	3.0 (2.0, 5.0)	3.0 (2.0, 4.0)	3.0 (2.0, 4.0)	3.0 (2.0, 5.0)	0.862
Maternal BMI (kg/m²)	20.5 (18.7, 22.9)	20.8 (19.1, 22.9)	21.0 (19.1, 22.8)	21.2 (19.3, 24.7)	0.255
AMH (ng/mL)	6.3 (3.4, 9.8)	6.9 (4.3, 10.6)	7.2 (4.2, 10.8)	5.6 (3.6, 8.7)	0.059
FSH (mIU/mL)	6.6 (5.8, 7.8)	6.5 (5.5, 7.6)	6.6 (5.5, 7.5)	6.2 (5.3, 8.2)	0.394
LH (mIU/mL)	5.6 (3.8, 8.5)	5.2 (3.7, 7.3)	5.4 (3.9, 7.7)	5.5 (4.0, 9.8)	0.321
E2 (pg/mL)	42.5 (31.0, 59.0)	43.0 (32.0, 54.0)	44.0 (33.0, 58.0)	46.0 (35.0, 59.0)	0.334
P (pg/mL)	0.6 (0.4, 0.9)	0.6 (0.39, 0.83)	0.6 (0.4, 0.8)	0.6 (0.4, 0.9)	0.886
Fertilization					< 0.01
IVF	81.5 (106/130)	83.9 (521/621)	90.5 (297/328)	96.9 (63/65)	
ICSI	18.5 (24/130)	16.1 (100/621)	9.5 (31/328)	3.1 (2/65)	
Endometrium thickness (mm)	9.0 (8.2, 10.2)	9.0 (8.3, 10.0)	9.0 (8.4, 10.0)	9.0 (8.6, 10.2)	0.498
Endometrial preparation (%)					0.072
Natural cycle	92.3 (120/130)	95.3 (592/621)	97.3 (319/328)	98.5 (64/65)	
HRT cycle	7.7 (10/130)	4.7 (29/621)	2.7 (9/328)	1.5 (1/65)	
High-quality blastocyst rate (%)	57.7 ^a^ (57/130)	87.8 ^b^ (545/621)	91.2 ^b^ (299/328)	92.3 ^b^ (60/65)	< 0.01
Frozen time (months), median (IQR)	3.0 (2.0,4.0)	3.0 (2.0,3.0)	3.0 (2.0,4.0)	3.0 (2.0,4.0)	0.206

Note: 1. *P* value indicates differences among the four groups. *P* < 0.05 indicates statistically significant difference in results

2. a, b: different superscript letters in the same row indicate significant differences between groups. According to Bonferroni correction for multiple comparisons, the difference was statistically significant at *P* < 0.008 (0.05/6)

The clinical outcomes of different D3 cell number groups are listed in Table 2. The LBR increased with the D3 cell number: 40.8%, 45.7%, 56.1%, and 64.6% for groups A, B, C, and D, respectively. The difference among the groups was statistically significant (*P* < 0.01). A *post hoc* power analysis based on a two-sided level of significance at 0.05 indicated that our study reached a power of 86% to detect an increase in the LBR from 45.7 to 56.1% and a power of 82% to detect an increase in the LBR from 45.7 to 64.6%. Similarly, the rate of clinical pregnancy increased with the increase in D3 blastomere number and was 55.4%, 54.8%, 66.5%, and 69.2% for groups A, B, C, and D, respectively (*P* < 0.01). However, the miscarriage rate decreased with the increase in D3 cell number and was 23.6%, 16.2%, 15.6%, and 4.4% for groups A, B, C, and D, respectively; nevertheless, no significant differences were detected (*P* = 0.055).

**Table 2:** Clinical outcomes of frozen-thawed SBT cycles grouped by D3 cell numbers

	Group A (*n* = 130)	Group B (*n* = 621)	Group C (*n* = 328)	Group D (*n* = 65)	*P* value
Clinical pregnancy rate (%)	55.4^ab^ (72/130)	54.8^a^ (340/621)	66.5^b^ (218/328)	69.2^ab^ (45/65)	< 0.01
Miscarriage rate (%)	23.6^a^ (17/72)	16.2^ab^ (55/340)	15.6^ab^ (34/218)	4.4^b^ (2/45)	0.055
Live birth rate (%)	40.8^a^ (53/130)	45.7^a^ (284/621)	56.1^b^ (184/328)	64.6^b^ (42/65)	< 0.01

Note: 1. *P* value indicates statistical differences among the four groups. *P* < 0.05 indicated a statistically significant difference

2. a, b: different superscript letters in the same row indicate significant differences between groups. According to Bonferroni correction for multiple comparisons, the difference was statistically significant at *P* < 0.008 (0.05/6)

The SBT cycles were analyzed using multivariate logistic regression. After adjusting for confounding factors, such as maternal age, duration of infertility, anti-Müllerian hormone (AMH), follicle stimulating hormone (FSH), frozen time, fertilization method, endometrium thickness, blastocyst quality, and D3 cell number, we observed that blastocyst quality and D3 cell number were independently associated with the LBR. Groups C (OR = 1.477, 95% CI: 1.124–1.941, *P* = 0.005) and D (OR = 2.000, 95% CI: 1.166–3.429, *P* = 0.012) showed significantly higher LBRs than group B (Table 3).

**Table 3:** Results of multiple regression analysis for live birth rates

	Odds ratio	95% CI		*P*-value
Maternal age (years)	0.998	0.956	1.042	0.916
Duration of infertility (years)	0.984	0.930	1.042	0.587
AMH	0.988	0.962	1.015	0.373
FSH	0.989	0.921	1.061	0.752
Frozen time				0.249
<6 months	1 (Reference)			
6-12months	1.111	0.656	1.884	0.695
>12months	1.808	0.893	3.662	0.100
Fertilization method^#^	0.812	0.573	1.150	0.240
Endometrium thickness (mm)	1.022	0.940	1.110	0.610
Blastocyst quality ^a^	0.517	0.359	0.746	<0.01
Day 3 cell number				0.005
≤ 7 cells	0.963	0.644	1.440	0.854
8–9 cells	1 (Reference)			
10–12 cells	1.477	1.124	1.941	0.005
≥ 13 cells	2.000	1.166	3.429	0.012

^a^ Reference category: high-quality blastocysts

^#^ Reference category: IVF

CI, confidence interval, IVF in vitro fertilization, ICSI intracytoplasmic sperm injection

## Discussion

Selecting embryos with the highest developmental potential for transfer is the key to improving clinical outcomes of IVF. Our results showed that in the first vitrified-warmed day 5 SBT cycles of patients < 35 years of age, the clinical pregnancy and LBRs increased with the increase in D3 cell number of the transferred blastocyst. This finding suggests that D3 cell number may be an effective indicator for predicting the clinical outcome of blastocyst transfer cycles.

The cell cycle duration is approximately 10–12 h [12]. By D3, ‘normal’ embryos are assumed to reach 7–9 cells, have the highest proportion of chromosomal euploidy, and exhibit the best clinical implantation potential [11]. However, a low number of cells in the embryo (< 8 cells) generally results in a prolonged cell cycle, developmental arrest, and cellular debris. Therefore, such embryos have reduced clinical implantation potential [13]. In this study, the high-quality blastocyst formation, clinical pregnancy, and LBRs were lower for the 3–7 cell embryo group than for those of the other three groups, consistent with previous research results [13, 14].

The effect of the ‘accelerated’ embryos on clinical outcomes has been controversial. Some studies reported that D3 embryos with higher cell numbers (> 8 cells) have poorer clinical outcomes [15, 16]. In 2015, Kroener et al. [17] reported a significant increase in the rate of chromosomal aneuploidy in embryos with D3 cell numbers > 9. However, their study had certain limitations; for instance, embryo biopsies were performed on D3 rather than on D5. The proportion of chromosomal chimerism is high in D3 embryos and they undergo self-repair mechanisms during blastocyst formation. Therefore, the chromosomal status of D3 cleavage-stage embryos is not equivalent to the chromosomal status after blastocyst formation. In addition, for patients < 35 years old, a D3 cell number > 13 was associated with a relatively low aneuploidy rate in this study [17].

A recent study retrospectively analyzed the data of 3543 patients who underwent vitrified-thawed SBT and reported comparable clinical pregnancy rate and LBR between the > 8 cell group and 8 cell group [14]. However, the overall development rate of the embryos was low; on D3, 60.7% of embryos had ≤ 6 cells, and only 3.33% of embryos had > 8 cells. Therefore, studies with large sample sizes are needed to confirm these findings.

‘Accelerated’ embryos reportedly have a high potential for successful clinical implantation. For instance, Kong et al. collected data from 799 embryos cultured in a time-lapse incubator and compared the effects of cell number, developmental time, and division behavior on embryonic developmental potential [13]. Their results showed that for embryos with normal cleavage patterns, developmental potential, implantation, and LBRs increased with cell number. Moreover, Tian et al. [7] found that the LBRs were significantly higher in patients transplanted with 10-cell embryos (OR 1.62, 95% CI 1.03–2.53; *P* = 0.035) and ≥ 11-cell embryos (OR 2.14, 95% CI 1.47–3.11; *P* < 0.001) than in those transplanted with 8-cell embryos. These results are consistent with those of our study.

A high development rate indicates a strong growth ability of an embryo. Such embryos can usually form blastocysts at stage 4 and above on day 5, and their ICM and TE cell number are relatively high. The morphological score of these blastocysts is similar to or even better than that of embryos with a moderate cleavage rate [18]. However, most centers still prefer blastocysts with 7–9 cells on D3 when selecting blastocysts for transfer, which may result in a potential waste of embryos and possibly prolong the time to pregnancy (TTP).

Moreover, abnormal cleavage behavior of direct division may increase the number of blastomeres. However, such chromosomally abnormal embryos often cannot undergo normal activation of the embryonic genome; thus, they cannot continue to develop to compaction and blastocyst stages [19]. Since the size of the fragments and blastomeres is similar on D3, embryologists may misjudge the fragments as blastomeres, thereby incorrectly identifying these embryos as ‘accelerated’ embryos. However, expectedly, the probability of these embryos developing into usable blastocysts is relatively low. Therefore, most embryos with direct division or a high degree of fragmentation can be excluded using the blastocyst culture technology.

Additionally, differences in culture conditions may influence embryo cleavage and affect embryonic metabolic activity. Kasterstein et al. showed that the cell number of embryos incubated under 5% O_2_ exceeded that of those grown under 20% O_2_ conditions [20]. Moreover, culture conditions and male factors can affect the duration of the synthetic phase (S phase) and ooplasmic maturity [21, 22]. Besides, IVF centers worldwide use various culture media and environments, which may account for the inconsistent conclusions of various studies conducted so far.

Setting the inclusion criteria in this study corrected bias as much as possible to reduce the interference of confounding factors. However, the limitations of our study include its retrospective design and the data source being limited to the Department of Reproductive Medicine, Women and Children’s Hospital, affiliated with Xiamen University. Therefore, our findings should be verified in a future prospective multicenter clinical study.

## Conclusions

In conclusion, during the first vitrified-warmed day 5 SBT cycle of patients < 35 years of age, the LBR and clinical pregnancy rate increased with an increase in cell number on D3. The clinical outcome of blastocysts formed from embryos with > 9 cells on D3 was better than that of blastocysts formed from embryos with 8–9 cells on D3. Therefore, in cases with the same blastocyst morphological score, those derived from > 9 cells on D3 can be preferentially selected for transplantation to reduce the TTP considerably. The findings of this study provide insights to optimize blastocyst grading and provide evidence for improving the reference indicator used for blastocyst selection.

## Data Availability

The data that support the findings of this study are available from the corresponding author Ping Li upon reasonable request.

## Abbreviations

AMH: Anti-Müllerian hormone
ART: Assisted reproductive technology
BMI: Body mass index
CI: Confidence interval
D3: Day 3
FSH: Follicle-stimulating hormone
HRT: Hormone replacement therapy
ICM: Inner cell mass
LBR: Live birth rate
OR: Odds ratio
PGT: Preimplantation genetic testing
SBT: Single blastocyst transfer
TE: Trophoectoderm
TTP: Time to pregnancy
